# Moving Beyond G‐CSF Mobilization—Learning From a 15‐Year Experience of Different Stem Cell Mobilization Regimens in Multiple Myeloma

**DOI:** 10.1002/cam4.71068

**Published:** 2025-07-16

**Authors:** Sumeet Mirgh, Bhausaheb Bagal, Sachin Punatar, Anant Gokarn, Nishant Jindal, Akanksha Chichra, Lingaraj Nayak, Sumathi Hiregoudar, Minal Poojary, Suryatapa Saha, Sarika Parab, Shashank Ojha, Prashant Tembhare, Nikhil Patkar, Sweta Rajpal, Gaurav Chatterjee, Libin Mathew, Papagudi Subramanian, Navin Khattry

**Affiliations:** ^1^ BMT Unit, Department of Medical Oncology Tata Memorial Centre, ACTREC Navi Mumbai India; ^2^ Homi Bhabha National Institute (HBNI) Mumbai India; ^3^ Department of Transfusion Medicine Tata Memorial Centre, ACTREC Navi Mumbai India; ^4^ Department of Hematopathology Tata Memorial Centre, ACTREC Navi Mumbai India

**Keywords:** bortezomib, cyclophosphamide, G‐CSF, multiple myeloma, plerixafor, stem‐cell mobilization

## Abstract

**Background:**

Stem‐cell mobilization in multiple myeloma is usually done with G‐CSF with or without Cyclophosphamide (Cy) based chemotherapy/Plerixafor. Pre‐clinical data suggest the role of proteasome inhibitors in mobilization. We previously reported that Bortezomib (Bort) when added to a Cy‐based regimen had a better stem‐cell yield. Consequent to favorable results with Bort + Cy‐G‐CSF, we used Bortezomib with G‐CSF too. Hence, four different mobilization regimens were used—Bort + G‐CSF (Group‐1); G‐CSF + Plerixafor (Group‐2); Bort – Cy‐G‐CSF (Group‐3); Cy + G‐CSF (Group‐4). We report here our 15‐year retrospective analysis of these 4 mobilization regimens.

**Objectives:**

Primary objective was to determine proportion of patients with CD34^+^ dose ≥ 5 × 10^6^/kg in first apheresis in various groups. Secondary objectives were to determine median CD34^+^ dose (×10^6^/kg) in first apheresis, total median CD34^+^ dose (×10^6^/kg) of all harvests and frequency of mobilization failure. Mobilization failure was defined as total CD34^+^ dose of < 2 × 10^6^/kg or abandoned harvest attempt at physician's discretion after anticipating a poor collection.

**Results:**

All consecutive patients with MM aged 18–65 years who underwent stem‐cell mobilization from September 2007–December 2022 were included. In an intention‐to‐treat analysis, a total 200 patients with 205 mobilization attempts were analysed. The median age of the cohort was 48 years. The percentage of patients who collected ≥ 5 × 10^6^ CD34^+^ cells/kg in the first apheresis was 26%, 53%, 69%, and 63% in Groups 1–4, respectively (*p* = 0.0001). The median CD34 yield in the first harvest (×10^6^/kg) was 3.62, 5.20, 6.04, and 6.05 in Groups 1–4, respectively (*p* = 0.00004). The median total stem‐cell dose collected (×10^6^/kg) was 5.73, 6.17, 9.14, and 8.23 in Groups 1–4, respectively (*p* < 0.00001). Mobilization failure rates were 7%, 3%, 0%, and 2%, respectively (*p* = NS).

**Conclusion:**

Cyclophosphamide‐based chemo‐mobilization regimens with or without Bortezomib have the advantage of higher total stem‐cell yield, while they are equivalent to G‐CSF + Plerixafor for harvest in a single apheresis. The addition of Bortezomib to Cyclophosphamide may help to increase stem cell yield.

## Graphical abstract

We analysed our 15‐year data of various stem‐cell mobilization regimens in myeloma. Cyclophosphamide‐based chemo‐mobilization regimens with or without Bortezomib have the advantage of higher total stem‐cell yield, while they are equivalent to G‐CSF + Plerixafor for harvest in a single apheresis. Bortezomib + GCSF is an inferior regimen for mobilization.

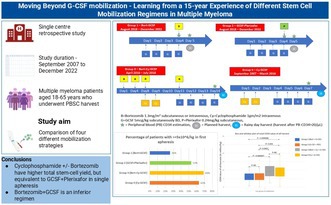

## 1 Introduction

Multiple myeloma (MM) accounts for 1.2% of all cancers in India, with an incidence of 1 per 100,000 [1]. An audit of all hematolymphoid malignancies at our hospital over a 5‐year period (2010–2014) showed that MM accounted for 10% of all hematological malignancies [2]. Lenalidomide‐based triplets (Bortezomib‐lenalidomide‐dexamethasone—VRd) [3, 4] or quadruplets [Daratumumab + VRd] [5, 6], followed by autologous stem‐cell transplant (ASCT) and lenalidomide maintenance, is standard of care treatment in newly diagnosed MM patients who are transplant eligible (TE). However, Lenalidomide is known to impair stem‐cell mobilization [7], which could impact the decision of ASCT. Additionally, even daratumumab has a negative impact on stem‐cell collection [8] with lesser yield and more need for plerixafor (PEF), in daratumumab‐based quadruplets versus triplets [9]. Hence, in the era of lenalidomide‐based triplets/quadruplets, there is a need to understand appropriate and cost‐effective mobilization strategies so that maximum TE patients can be consolidated with ASCT.

Hematopoietic stem cells (HSCs) are usually mobilized to peripheral blood (PB) using G‐CSF (Granulocyte‐colony stimulating factor) with chemotherapy (chemo‐mobilization) or without chemotherapy (steady state mobilization). Cyclophosphamide (Cy) based chemo‐mobilization offers advantages in terms of higher HSC yield [10] and can overcome the adverse impact of prior lenalidomide therapy on harvest [11]. However, there is a need for hospitalization and the risk of febrile neutropenia during chemo‐mobilization [12]. PEF (inhibits CXCR4‐SDF‐1 interaction) + G‐CSF was tested in a phase‐3 randomized trial versus G‐CSF alone. The PEF arm showed a higher stem‐cell yield with fewer apheresis sessions, which led to its approval [13]. Hence, steady‐state mobilization with G‐CSF + PEF has become increasingly popular [12]. Until a few years back, PEF was prohibitively expensive, thereby limiting its use in India. Even in the West, it could not be used for all patients, and its use had to be risk‐adapted in order to make it cost‐effective [14]. The generic version of PEF called Mozifor was first launched in 2014 by Hetero Drugs Ltd., India. The cost of PEF [Mozifor (Hetero Drugs Ltd., India) 2014–2018: INR 79,200 (equivalent to USD 1050), 2019–2020: INR 39,000 (equivalent to USD 520); Stemfor (Intas Drugs Ltd., India) 2021–2022: INR 36,540 (equivalent to USD 500)] has declined over the last decade. However, this still accounts for approximately 10% of the total cost of ASCT in India [15]. Besides, even with G‐CSF + PEF, mobilization failure rates of 7%–10% have been reported from India [16, 17]. This prompted a search for alternative strategies for mobilization, which are effective and feasible in the Indian setting.

Ghobadi et al. reported in 2014 from mouse models that proteasome inhibitors (PI) aid in HSC mobilization by down‐regulating VLA‐4 on bone marrow stromal cells, which interacts with VCAM‐1 on HSC [18]. The same group showed in 2018 a similar effect of Ixazomib on HSC mobilization, with a benefit intermediate between G‐CSF and PEF [19]. This favorable effect of PIs, coupled with a potential tumor purging effect, is highly desirable. Moreover, Bortezomib (Bort) is much cheaper (INR 2500, equivalent to USD 30) than PEF in India. Hence, consequent to their first report in 2014, we used Bort with Cy chemo‐mobilization, and published that Bort+Cy‐G‐CSF (vs Cy‐G‐CSF) showed a trend towards higher CD34 yield and lesser apheresis sessions without additional toxicity [20]. Considering the benefit of Bort with chemo‐mobilization, we subsequently used Bort along with G‐CSF (Bort‐G‐CSF) for mobilization. Hence, over the last two decades, different mobilization strategies evolved in our institution—initially, Cy + G‐CSF, followed by Bort‐Cy‐G‐CSF. Introduction of cheaper forms of generic PEF in 2018 made it economically feasible for use. Other mobilization regimens were—G‐CSF + PEF and Bort+G‐CSF. There is limited data comparing mobilization regimens, especially from low‐middle income countries where HSC collection may not be done after 4–6 cycles of first‐line therapy. We hereby report our 15‐year experience of various mobilization regimens for MM.

## 2 Materials and Methods

All patients with MM aged 18–65 years who underwent peripheral blood stem‐cell (PBSC) mobilization between 1st September 2007—31st December 2022 were included in this single centre retrospective analysis. MM patients who were planned for ASCT, as consolidation therapy, either upfront or at relapse were included. Patients of other plasma cell dyscrasias viz. Amyloidosis, POEMS syndrome were excluded.

### 2.1 Mobilization Regimens

**FIGURE 1:**
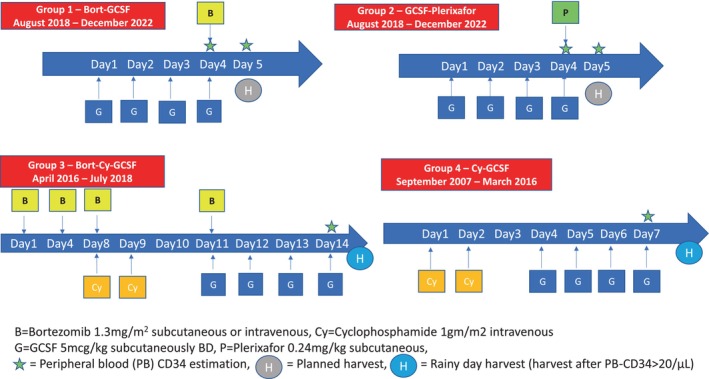
Schema of different mobilization regimens as per respective time‐periods.

Various mobilization regimens [Group‐1—Bort‐G‐CSF (August 2018–December 2022); Group‐2—G‐CSF‐PEF (August 2018–December 2022); Group‐3—Bort–Cy‐G‐CSF (April 2016–July 2018); Group‐4—Cy‐G‐CSF (September 2007–March 2016)] were used as per time periods mentioned in Figure 1. Our institutional practice has always been to collect a CD34 cell dose of 5 million/kg which would suffice for two transplants.

#### 2.1.1 Group‐1 (Bort + G‐CSF)

G‐CSF (5 mcg/kg subcutaneously BD) was given on Days 1–4, and the 5th day morning prior to harvest. Bort (1.3 mg/m^2^ intravenous or subcutaneous) was given on Day‐4 evening, 14 h prior to planned apheresis. G‐CSF was administered on an out‐patient basis for the first 3 days, and patients were admitted on Day‐4 followed by PBSC harvest as in‐patient on Day‐5. CD34 monitoring was done on Day‐4, and prior to harvest on Day‐5 (and Day‐6, if harvest was planned for Day‐6). If PB‐CD34 was less than 20/μL on Day 4, PEF was used according to the physician's discretion (“Pre‐emptive” PEF).

#### 2.1.2 Group‐2 (G‐CSF + PEF)

G‐CSF (5 mcg/kg subcutaneously BD) was given on Days 1–5. PEF (0.24 mg/kg subcutaneously) was given on Day‐4 evening 11 h prior to planned apheresis. G‐CSF was administered on an out‐patient basis for the first 3 days, and patients were admitted on Day‐4 followed by PBSC harvest as in‐patients on Day‐5. CD34 monitoring was done on Day‐4, and prior to harvest on Day‐5 (and Day‐6, if harvest was planned for Day‐6).

In Groups 1 and 2, planned apheresis was done on Day 5, and additionally on Day 6, if the first harvest was inadequate (< 5 million CD34 cells/kg).

#### 2.1.3 Group‐3 (Bort‐Cy‐G‐CSF)

Bort (1.3 mg/m^2^ intravenous) was given on Days‐1,4,8,11, with Cy (1 g/m^2^ intravenous) on Days 8 and 9, followed by G‐CSF (5 mcg/kg subcutaneously BD) from Day 11 onwards, similar to Niesvizky et al. [21]. PB‐CD34 monitoring was started from Day 14 (72 h after the first dose of G‐CSF) and continued daily until apheresis. Patients were admitted the evening prior to scheduled Cy. Cy was given as an in‐patient (along with Bort on Day‐8) and patients were discharged 24 h after the second dose of Cy. Bort on Days 1, 4, and 11 was given on an out‐patient basis. Patients were re‐admitted on Day 13 (evening prior to first CD34 enumeration) or earlier in case of fever, until harvest.

#### 2.1.4 Group‐4 (Cy‐G‐CSF)

In group‐4, Cy (1 g/m^2^) was given on Day 1 and 2, followed by G‐CSF (5 mcg/kg subcutaneously BD) from Day‐4 onwards. PB‐CD34 monitoring was started from Day‐7 (72 h after the first dose of G‐CSF) and continued daily until apheresis. Patients were admitted an evening prior to scheduled Cy. Cy was given as an in‐patient and patients were discharged 24 h after the second dose of Cy. They were re‐admitted on Day‐6 (evening prior to first CD34 enumeration) or earlier in case of fever, until harvest.

Stem‐cell harvest was done in groups 3 and 4 once peripheral blood CD34 (PB‐CD34) was > 20/μL. In groups 3 and 4, if PB‐CD34 was < 20/μL for 2 subsequent days, PEF was used according to physician's discretion. PEF was used in any group after the first harvest if the cell dose was deemed inadequate (“Therapeutic” or “Salvage” PEF).

In both the Cy cohorts, subjects received anti‐emetic prophylaxis (intravenous granisetron, dexamethasone) and intravenous hydration (0.9% normal saline 2 L/m^2^), along with MESNA (Sodium 2‐Mercaptoethane sulfonate) 1 g/m^2^ over 12 h BD on the days of Cy to prevent hemorrhagic cystitis.

In patients with poor peripheral venous access, a double‐lumen high‐flow hemodialysis catheter (11.5 French Mahurkar) was inserted for stem‐cell collection. In all groups, serum electrolytes (sodium, potassium, chloride, calcium, magnesium, and phosphate) were checked 4 h post‐apheresis, and patients were discharged after electrolyte correction.

### 2.2 Stem Cell Harvest, and Cryopreservation

PBSC harvest was done using COBE spectra (Terumo BCT) or Amicus (Fresenius Kabi) cell separation platforms, as per manufacturer's instructions. Anticoagulation with acid‐citrate‐dextrose (ACD) with blood:ACD ratio of 10:1–12:1 was used. Oral or intravenous calcium gluconate was given during apheresis to prevent hypo‐calcaemic symptoms due to citrate. It was ensured that hematocrit was at least 25% on the day of apheresis. Usually, a total of four times total blood volume was processed. CD34 dose of 2 × 10^6^/Kg patient's body weight was considered minimum, while 5 × 10^6^/Kg body weight was deemed adequate. If adequate CD34 dose was not achieved on first day of harvest, then apheresis was repeated on subsequent days (maximum of four apheresis sessions). PBSC product was analysed for total leukocyte counts, mononuclear cell count, and CD34+ stem‐cell enumeration at the end of apheresis. CD34 enumeration was performed through single‐platform multi‐color flow cytometry following the International Society of Hematotherapy and Graft Engineering (ISHAGE) guidelines. In summary, this method involved a sequential Boolean gating strategy utilizing a combination of CD45 and CD34, along with the incorporation of beads for absolute quantification. All harvested PBSCs were cryopreserved within 24 h of apheresis with 8.7% DMSO (Di‐Methyl‐Sulfoxide) in a 1:1 ratio (DMSO concentration 4.35% in the final product), and stored at −80°Celsius using uncontrolled rate freezing in mechanical freezers.

### 2.3 ASCT—Transplant Procedure and Supportive Care

Patients received Melphalan (140–200 mg/m^2^) conditioning followed by PBSC infusion after 24 h. Prior to infusion, PBSC bags were thawed in a water bath at 37°C, and their viability was checked with the trypan‐blue dye exclusion method. All patients received prophylactic G‐CSF from Day +5, along with fungal (Voriconazole) (from Day 0) and viral (Aciclovir) (from Day 0) prophylaxis, as per institutional policy. Platelet and myeloid engraftment were defined as per standard criteria. Engraftment syndrome was diagnosed as per Spitzer criteria [22].

### 2.4 Objectives

Primary objective was to determine the proportion of patients with CD34^+^ dose ≥ 5 × 10^6^/kg in the first apheresis in various groups. Secondary objectives were to determine total median CD34^+^ dose (×10^6^/kg) of all harvests, median CD34^+^ (×10^6^/kg) in the first apheresis, and the incidence of mobilization failure. Mobilization failure in an attempt was defined as total CD34^+^ dose of < 2 × 10^6^/kg or abandoned harvest attempt anticipating a poor collection. Analysis was done as per the planned regimen [intention‐to‐treat (ITT) analysis]. In order to rule out the added benefit of plerixafor, an additional plerixafor subtraction analysis was done. Subgroup analysis was done for patients who received VRd induction prior to harvest, and mobilization efficacy in patients who received prior radiotherapy (RT) and > 4 cycles of lenalidomide.

### 2.5 Data Collection

Clinical parameters [age, gender, lines of therapy before harvest, chemotherapy regimen(s) received, lenalidomide exposure and duration, baseline International Staging system (ISS), disease status before harvest], PBSC parameters (PB‐CD34 on day of harvest, PEF use—pre‐emptive/therapeutic, CD34 dose collected in first apheresis, total CD34 dose, number of apheresis days, mobilization failure) and transplant characteristics [patients who underwent ASCT, date of transplantation, median day of neutrophil and platelet engraftment, engraftment syndrome, median progression‐free survival (PFS) and overall survival (OS)] were analyzed and compared in all four groups.

### 2.6 Statistical Methods

Data was tabulated in a Microsoft‐Excel sheet and analysed using SPSS v23.0. Qualitative data were compared using the chi‐square test, and quantitative data by the Kruskal‐Wallis test. For small frequencies, Fisher's exact test was used. Box and Whisker plots were used to compare CD34 dose among all four groups of mobilization. Survival analysis was done by the Kaplan–Meier method. Reported *p* values were two sided, and *p*‐value < 0.05 was considered statistically significant. Data of all patients was updated till 31st December 2023. Follow‐up was defined as the duration between the date of diagnosis and the date of last follow‐up or date of death.

## 3 Results

### 3.1 Baseline Characteristics

A total of 205 mobilization attempts were made in 200 patients. Median age of the study cohort was 48 years (Range: 27–65 years), and 143 (70%) patients were male. Baseline patient characteristics are shown in Table 1. Among the four groups—43 were in Group‐1 (Bort+G‐CSF), 30 in Group‐2 (G‐CSF + PEF), 42 in Group‐3 (Bort‐Cy‐GCSF), 90 in Group‐4 (Cy‐GCSF). There was no difference in the median age and ISS staging distribution in all four groups. The most common induction regimen used in the first two groups (Bort + G‐CSF; G‐CSF + PEF) was VRd, as compared to VCd in the latter two groups (Bort‐Cy‐G‐CSF; Cy‐G‐CSF). More patients received lenalidomide in the first two groups (Bort + G‐CSF; G‐CSF + PEF), while there was no difference in the median duration of lenalidomide exposure in the ones who received it in the four groups. Median follow‐up of the entire cohort was 64 months.

**TABLE 1:** Baseline patient characteristics in four groups.

	Group 1—Bort‐G‐CSF	Group 2—G‐CSF‐Plerixafor	Group 3—Bort‐Cy‐G‐CSF	Group 4—Cy‐G‐CSF	*p*
Time‐period	August 2018—December 2022	August 2018—December 2022	April 2016—July 2018	September 2007—March 2016	
Number of patients	43	30	42	90	
Male/female (*n*)	26/17	24/6	31/11	62/28	NS
Median age in years (range)	47 (29–62)	48 (28–63)	47 (27–63)	49 (31–65)	NS
ISS‐1; *n* (%)	14 (33%)	7 (23%)	10 (24%)	25 (28%)	NS
ISS‐2; *n* (%)	12 (30%)	7 (23%)	9 (22%)	25 (28%)	
ISS‐3; *n* (%)	14 (33%)	10 (33%)	11 (27%)	34 (38%)	
Missing; *n* (%)	1 (3%)	6 (20%)	12 (28%)	6 (6%)	
**Baseline induction** ^b^					
VCd; *n* (%)	13 (30%)	7 (23%)	30 (71%)	44 (49%)	< 0.00001
VRd; *n* (%)	27 (63%)	18 (60%)	17 (40%)	15 (17%)	
VTd; *n* (%)	2 (5%)	2 (7%)	2 (5%)	8 (9%)	
Rd.; *n* (%)	0	0	1 (3%)	8 (9%)	
Others^a^; *n* (%)	1 (2%)	3 (7%)	0	16 (18%)	
Median lines of prior treatment; (range)	1 (1–2)	1 (1–3)	1 (1–2)	1 (1–3)	NS
First remission at time of harvest; *n* (%)	38 (88%)	20 (66%)	28 (63%)	58 (64%)	0.03
Len exposure prior to harvest; *n* (%)	32 (74%)	21 (70%)	22 (53%)	23 (26%)	< 0.00001
Median duration of Len exposure; months (IQR)	5 (4–6)	6 (4–10)	4 (3–8)	6 (4–6)	NS
CR/VGPR at time of harvest; *n* (%)	40 (93%)	24 (80%)	30 (72%)	54 (60%)	0.0007
Receipt of prior radiotherapy; *n* (%)	9 (21%)	9 (30%)	16 (38%)	27 (30%)	NS
Median time from diagnosis to harvest; months (IQR)	7.5 (6–9)	11 (7–18)	8 (5–12)	7 (5.5–12)	0.07

Abbreviations: Bort = Bortezomib, CR = Complete response, Cy = Cyclophosphamide, G‐CSF = Granulocyte colony stimulating factor, ISS = International Staging System, Len = Lenalidomide, Rd. (Lenalidomide‐dexamethasone), VCd (Bortezomib‐Cyclophosphamide‐dexamethasone), VGPR = Very good partial response, VRd (Bortezomib‐lenalidomide‐dexamethasone), VTd (Bortezomib‐thalidomide‐dexamethasone).

^a^ Others—CTd (Cyclophosphamide‐Thalidomide‐Dexamethasone), CDd (Cyclophosphamide‐liposomal doxorubicin‐dexamethasone), Vd (Bortezomib‐dexamethasone), Td (Thalidomide‐dexamethasone), VAD (Vincristine‐Adriamycin‐dexamethasone).

^b^ Values in some columns may exceed 100%, as some patients received more than one line of therapy prior to harvest.

### 3.2 First Apheresis, Total Apheresis CD34 Cell‐Dose and Mobilization Failure

**FIGURE 2:**
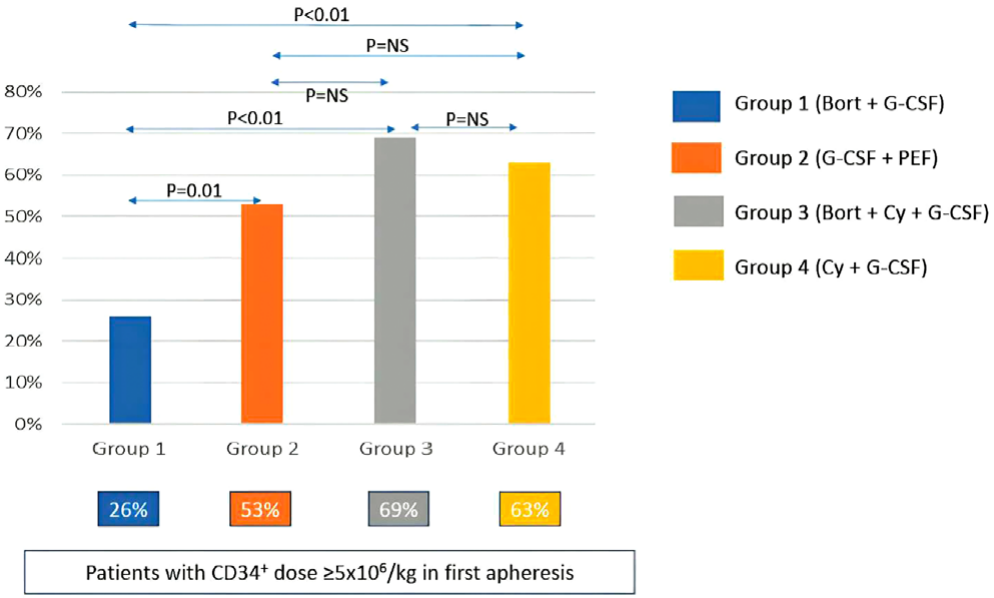
Proportion of patients who collected ≥ 5 × 10^6^/kg in first apheresis.

Percentage of patients who collected ≥ 5 × 10^6^ CD34^+^ cells/kg in the first apheresis were 26%, 53%, 69%, and 63%, respectively, in Groups 1 (Bort + G‐CSF), 2 (G‐CSF + PEF), 3 (Bort‐Cy‐G‐CSF) and 4 (Cy‐G‐CSF) (*p* = 0.0001) (Figure 2). The median day of first collection in Groups 3 (Bort‐Cy‐G‐CSF) and 4 (Cy‐GCSF) was Day 17 (Range: Day 15–Day 19) and Day 10 (Range: Day 9–Day 16), respectively.

**FIGURE 3:**
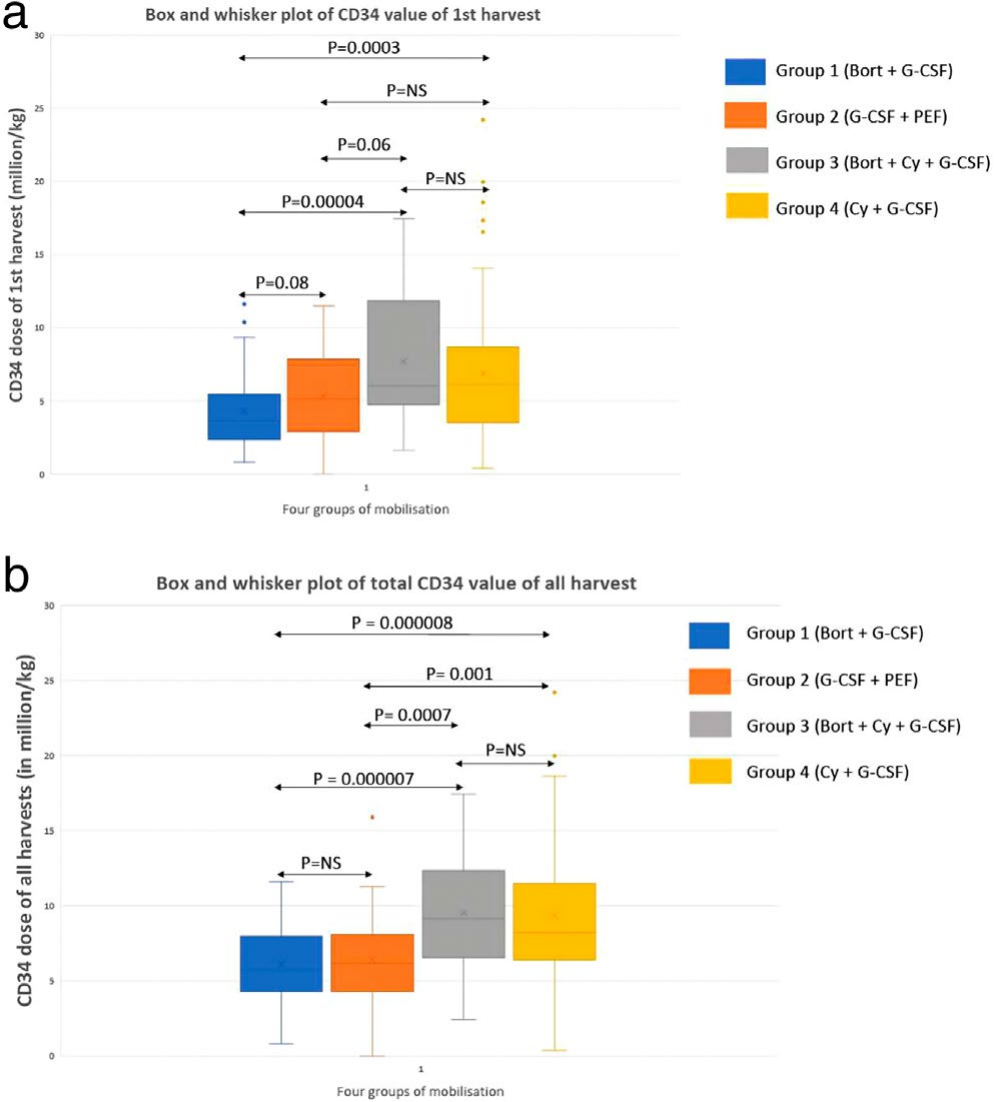
(a) (upper panel)—Box and Whisker plot of CD34 cell‐dose in first apheresis. (b) (lower panel)—Box and Whisker plot of CD34 cell‐dose in all apheresis.

CD34+ cell dose collected in the first apheresis and all harvests is shown in Table 2. First apheresis and total CD34 yield amongst all four groups is compared in Figure 3. For first apheresis yield, in comparison to group‐1 (Bort + G‐CSF), both groups 3 (Bort‐Cy‐G‐CSF) and 4 (Cy‐G‐CSF) fared significantly better (Group‐3 vs. Group‐1; *p* = 0.00004; Group‐4 vs. Group‐1; *p* = 0.0003), while there was a trend to significance for group‐2 (G‐CSF + PEF) (Group‐2 vs. Group‐1; *p* = 0.08). Importantly, first harvest CD34 yield in Group‐2 (G‐CSF + PEF) was comparable to both the Cy groups (Group‐2 vs. Group‐3; *p* = 0.06; Group‐2 vs. Group‐4; *p* = NS). For total CD34 yield, both Cy groups [Group‐3 (Bort‐Cy‐G‐CSF), 4 (Cy‐G‐CSF)] had significantly higher total CD34 dose as compared to the first two (Bort + G‐CSF; G‐CSF + PEF) groups (Group‐3 vs. Group‐1; *p* = 0.000007; Group‐4 vs. Group‐1; *p* = 0.000008; Group‐3 vs. Group‐2; *p* = 0.0007; Group‐4 vs. Group‐2; *p* = 0.001). However, there was no difference between the first two (Bort + G‐CSF vs. G‐CSF + PEF) groups for total CD34 dose.

**TABLE 2:** Comparison of CD34 stem cell yield in all four groups.

	Group 1—Bort‐GCSF (*n* = 43)	Group 2—GCSF‐Plerixafor (*n* = 30)	Group 3—Bort‐Cy‐GCSF (*n* = 42)	Group 4—Cy‐GCSF (*n* = 90)	*p*
Patients who collected ≥ 5 million in 1st harvest; *n* (%)	11 (26%)	16 (53%)	29 (69%)	57 (63%)	0.0001 (Group 1 vs. 3)
CD34 cell dose in 1st harvest (×10^6^/kg); median (range)	3.62 (0.81–11.61)	5.20 (1.54–11.47)	6.04 (1.6–17.44)	6.05 (0.4–24.2)	0.00004 (Group 1 vs. 4)
Total CD34 cell dose collected (×10^6^/kg); median (range)	5.73 (0.81–11.61)	6.17 (1.79–15.9)	9.143 (2.42–17.44)	8.237 (0.4–24.2)	< 0.00001 (Group 1 vs. 3)
Median number of apheresis sessions; (range)	2 (1–2)	1 (1–2)	1 (1–2)	2 (1–4)	NS
Mobilization failure; *n* (%)	3 (7%)	1 (3%)	0	2 (2%)	NS

Abbreviations: Bort = Bortezomib, Cy = cyclophosphamide, GCSF = granulocyte colony stimulating factor, NA = not applicable, NS = not significant, PB CD34 = peripheral blood CD34, PEF = plerixafor.

There was no difference in the incidence of mobilization failure and median number of apheresis sessions in four groups (Table 2). More patients in groups 1 (Bort + G‐CSF) and 4 (Cy + G‐CSF) [Bort + G‐CSF (60%) vs. G‐CSF + PEF (36%) vs. Bort‐Cy‐G‐CSF (40%) vs. Cy‐G‐CSF (57%); *p* = 0.07] required more than 1 apheresis. Maximum apheresis sessions for a patient in one mobilization attempt were four (Group‐4: Cy + G‐CSF). Four patients in Group‐1 (Bort + G‐CSF) and one patient in Group‐4 (Cy + G‐CSF) underwent a second mobilization attempt with G‐CSF‐PEF (Group‐2) in view of inadequate harvest (< 5 million) in the first attempt.

### 3.3 Plerixafor (PEF) use (Refer Table 3)

**TABLE 3:** Use of plerixafor in different groups of mobilization.

	Group 1—Bort‐GCSF (*n* = 43)	Group 2—GCSF‐Plerixafor (*n* = 30)	Group 3—Bort‐Cy‐GCSF (*n* = 42)	Group 4—Cy‐GCSF (*n* = 90)	*p*
PB CD34 (/μL) on the day of 1st harvest; median (range)	48.45 (8.98–257.3)	59.2 (2.1–217.8)	60.66 (16.4–170.7)	55.11 (11.2–295.13)	NS
PB CD34 (/μL) 1 day prior to 1st harvest; median (range)	21.12 (1.93–148.08) [*n* = 43]	12.76 (0.8–137) [*n* = 29]	12.58 (1.75–55.33) [*n* = 41]	17.01 (3.96–78.9) [*n* = 82]	0.008 (Group 1 vs. 3)
Patients with PB CD34 < 20/μL 1 day prior to harvest; *n* (%)	20/43 (47%)	19/29 (63%)	32/41 (78%)	57/84 (68%)	0.02 (Group 1 vs. 3)
Patients who received PEF in view of PB CD34 < 20/μL 1 day before harvest; *n* (%) (A)	15/20 (75%)	NA	9/32 (28%)	3/57 (5.5%)	< 0.0001 (Group 1 vs. 4)
PEF given after 1st day harvest in whole cohort; *n* (%) (B)	15/43 (35%)	11/30 (37%)	0/42 (0%)	3/90 (3%)	0.012 (Group 1 vs. 3)
Total PEF used (A + B); *n* (%)	20 (47%)	25 (83%)	9 (22%)	6 (7%)	< 0.00001 (Group 2 vs. 4)

Abbreviations: Bort = bortezomib, Cy = cyclophosphamide, GCSF = granulocyte colony stimulating factor, NA = not applicable, NS = not significant, PB CD34 = peripheral blood CD34, PEF = plerixafor.

PEF was used either “pre‐emptively” or as “salvage”, as described earlier. Apart from Group‐2 (G‐CSF + PEF), nearly half (47%) mobilization attempts in group‐1 (Bort + G‐CSF), versus 22% in group‐3 (Bort‐Cy‐G‐CSF), and 7% in group‐4 (Cy‐G‐CSF) required PEF. In groups‐1 (Bort + G‐CSF), 2 (G‐CSF + PEF), 3 (Bort‐Cy‐G‐CSF) and 4 (Cy‐G‐CSF), 35%, 37%, 0%, and 3% required PEF for salvage, respectively. The proportion of patients with PB‐CD34 < 20/μL 1 day prior to apheresis in the four groups was 47%, 63%, 78%, and 68%, respectively. The median PB‐CD34 1 day prior to apheresis in the four groups was 21.12, 12.76, 12.58, and 17.01/μL, respectively. The median PB CD34 on the first day of harvest was comparable in all four groups [Group‐1 (Bort + G‐CSF) 48.4/μL, vs. Group‐2 (G‐CSF + PEF) 59.2/μL, vs. Group‐3 (Bort‐Cy‐G‐CSF) 60.6/μL, vs. Group‐4 (Cy‐G‐CSF) 55.1/μL; *p* = NS] (Table 3).

### 3.4 Plerixafor (PEF) Subtraction Analysis—Analysis of Patients Who Did Not Receive PEF (Table S1)

In order to see the additional benefit of Bort or Cy chemotherapy to G‐CSF, an analysis of mobilization attempts wherein PEF was not given was done in Groups 1 (Bort + G‐CSF), 3 (Bort‐Cy‐G‐CSF) and 4 (Cy‐G‐CSF). In groups 1, 3, and 4, 39% (Group‐1; 9/23), 70% (Group‐3; 23/33), and 64% (Group‐4; 54/84) collected ≥ 5 million/kg in 1st apheresis (*p* = 0.048), respectively. Median CD34 cell dose (in million/kg) harvested in 1st apheresis was 4.77 (Group‐1) vs. 6.1 (Group‐3) vs. 6.12 (Group‐4) [*p* = 0.02], while total CD34 cell dose collected was 6.03 (Group‐1) vs. 9.86 (Group‐3) vs. 8.58 (Group‐4) [*p* = 0.00007].

### 3.5 Subgroup Analysis

#### 3.5.1 Impact of Prior Radiotherapy (RT) and Lenalidomide

Since G‐CSF + PEF arm had a non‐inferior stem‐cell yield in 1st apheresis as compared to both the Cy groups (Bort‐Cy‐G‐CSF and Cy‐G‐CSF) (Figure 3), we looked at harvest characteristics in this group amongst those who received RT (to sites of hematopoiesis, i.e., vertebrae or pelvis) and more than four cycles of lenalidomide. CD34 yield in patients who received prior RT and lenalidomide > 4 cycles were much lesser in the first two groups than both the Cy groups. Group‐3 (Bort‐Cy‐G‐CSF) had the highest CD34 yield in first harvest in patients with prior RT and lenalidomide > 4 cycles (Table S5).

#### 3.5.2 Comparison of Mobilization Strategies in Patients Who Received VRd Induction Prior to Harvest

In patients who received VRd prior to harvest, the frequency of patients who collected a CD34 cell dose of > 5 million/kg in the four groups were 23%, 56%, 59%, and 81%, respectively. The median first harvest CD34 cell dose (in million/kg) was 3.62, 5.76, 5.61, and 5.72 in the four groups, respectively. The total CD34 cell dose (in million/kg) was 6.05, 6.7, 6.8, and 8.8 in the four groups, respectively. Mobilization failures failure rates were 11%, 5%, 0%, and 6%, respectively.

#### 3.5.3 Group‐1 (Bort‐G‐CSF) – CD34 Yield With Intravenous Versus Subcutaneous Bortezomib

Subgroup analysis within group‐1 (per‐protocol) showed that intravenous Bort (*n* = 16) was better than subcutaneous Bort (*n* = 19) for median CD34 collection (in million/kg) in first apheresis (4.97 vs. 3.08; *p* = 0.05) and total harvests (7.54 vs. 5.21; *p* = 0.09), respectively.

A separate analysis of increment in PB‐CD34 from 1 day prior and on the day of first harvest, and per‐protocol analysis has been described in supplemental appendix.

### 3.6 Increment in PB‐CD34 from one day prior and on the day of first harvest

**FIGURE 4:**
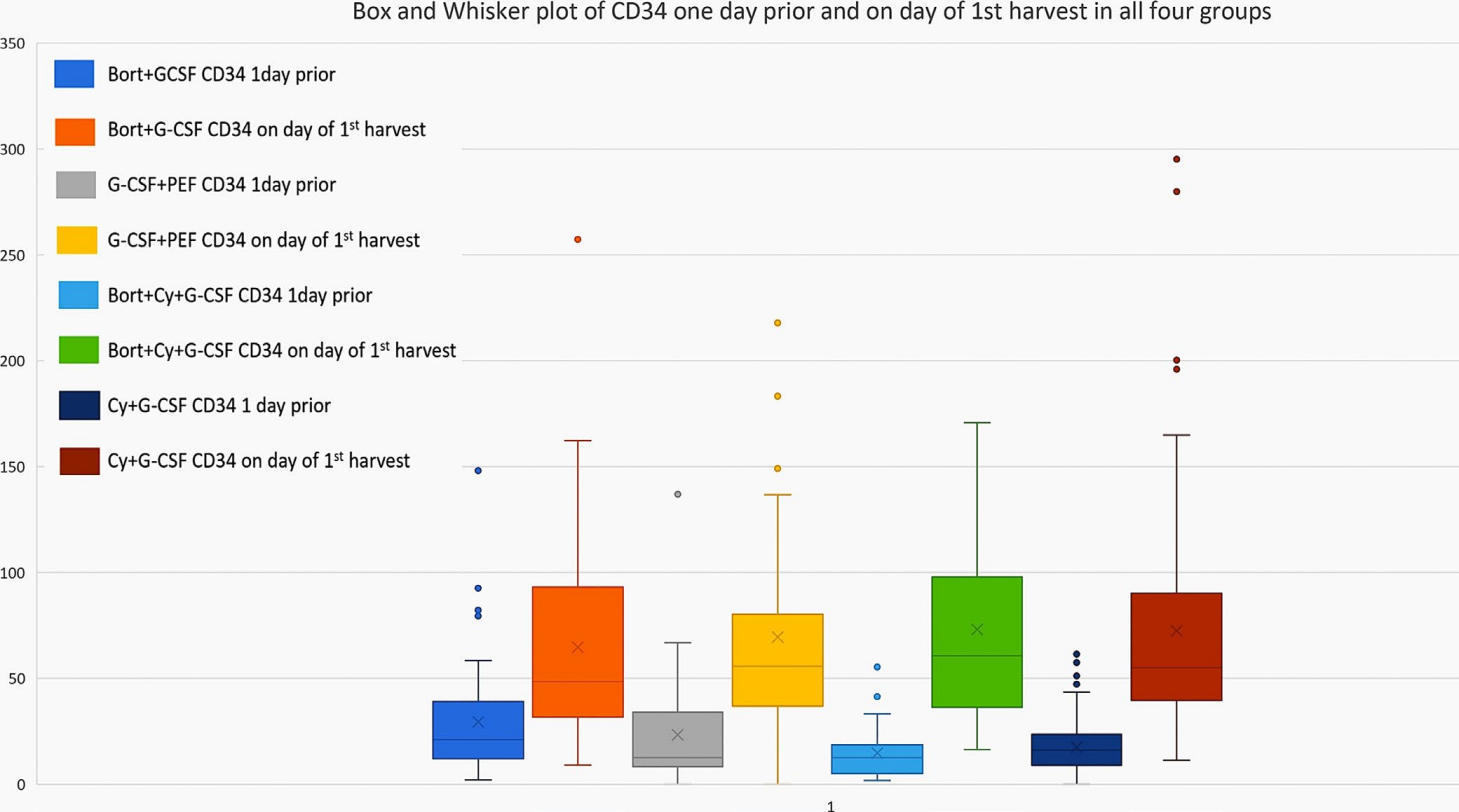
Box and Whisker plot of peripheral blood CD34 value 1 day prior and on the day of first apheresis.

The difference between median PB‐CD34 values one day prior (pre‐CD34) and on day of first harvest prior to apheresis were Group‐1 (Bort+G‐CSF) 28.72/μL (range: 0–114.5), Group‐2 (G‐CSF+PEF) 38.12/μL (range: 1.3–151.03), Group‐3 (Bort‐Cy‐G‐CSF) 38.27/μL (range: 4.34–168.95), Group‐4 (Cy‐G‐CSF) 35.55/μL (range: −1.29 to 254.9) [*p* = NS]. In comparison to pre‐CD34, this translates to 1.35‐fold, 2.98‐fold, 3.04‐fold, and 2.08‐fold increment in CD34 values in the four groups, respectively (Figure 4).

### 3.7 ASCT Outcomes

Amongst 200 patients, 176 (88%) underwent ASCT. Importantly, there was no difference in the median day of neutrophil (12 vs. 11 vs. 11.5 vs. 11; *p* = NS) and platelet (12 vs. 12 vs. 13.5 vs. 12; *p* = NS) engraftment, and the incidence of engraftment syndrome (37% vs. 33% vs. 34% vs. 32%; *p* = NS) between the four groups (Table 4). Median PFS and OS of the entire cohort post‐ASCT was 46 months and not reached, respectively (Figure S1).

**TABLE 4:** Outcomes of patients who underwent ASCT (autologous stem cell transplant).

	Group 1—Bort‐GCSF (*n* = 43)	Group 2—GCSF‐Plerixafor (*n* = 30)	Group 3—Bort‐Cy‐GCSF (*n* = 42)	Group 4—Cy‐GCSF (*n* = 90)	*p*
Patients who underwent ASCT; *n* (%)	28 (65%)	24 (80%)	38 (91%)	87 (97%)	< 0.00001 (Group 1 vs. 4)
*ASCT in*					
First remission	24 (85%)	15 (64%)	24 (63%)	55 (63%)	NS
Relapse	4 (15%)	9 (36%)	14 (37%)	33 (37%)	
Median day of neutrophil engraftment	12	11	11.5	11	NS
Median day of platelet engraftment	12	12	13.5	12	NS
Engraftment syndrome	11 (39%)	8 (33%)	13 (34%)	28 (32%)	NS
Median PFS (months)	Not reached	35	63	39	NS
Median OS (months)	Not reached	126	Not reached	Not reached	NS
Median follow‐up (months)	53	54	78	87	0.004

Abbreviations: ASCT = autologous stem cell transplant, OS = overall survival, PFS = progression‐free survival.

## 4 Discussion

In this study, we have evaluated different PBSC mobilization strategies, including conventional Cy chemo‐mobilization and G‐CSF‐PEF mobilization, both with and without Bortezomib. While strategies for PBSC mobilization have evolved over time, its objectives stay unchanged, i.e., efficient stem‐cell collection with minimum mobilization failure.

There are multiple studies reporting the comparison of Cy + G‐CSF versus G‐CSF ± PEF [10, 23, 24, 25, 26, 27]. However, the majority of these have a small sample size, and only three of them have more than 100 patients [23, 24, 25]. In contrast to others [10, 23, 24, 25, 26, 27], the median age of our cohort (48 years) was one decade younger with a higher proportion of ISS‐III (38%; *n* = 69/180) patients. This is similar to other data from India [2, 28, 29]. Our data is comparable with others [10, 24, 26] with respect to lenalidomide exposure prior to harvest. However, the median duration of lenalidomide exposure was 1–2 cycles more in our cohort, as compared to the literature [10, 24, 26]. Inspired by pre‐clinical data of Ghobadi et al. [18], small single‐arm studies have described the benefit of Bort addition to Cy‐G‐CSF [21] and G‐CSF [30]. Similar to our report [20], Ohno et al. from Japan described the benefit of Bort‐Cy‐G‐CSF over Cy‐G‐CSF; however, their study had 15 and 18 patients in each arm, respectively [31]. However, none of these studies have systematically compared Bort‐based mobilization strategies (Bort‐G‐CSF and Bort‐Cy‐G‐CSF) with conventional regimens (G‐CSF + PEF and Cy‐G‐CSF).

The primary objective varies in different studies—stem‐cell dose of 3 million/kg [10], 4 million/kg [23, 25, 27], or 5 million/kg [24, 26]. We considered 5 million, as that cell dose is sufficient for two transplants. With a 5 million/kg target, in spite of having a relatively younger population, CD34 in our Cy‐G‐CSF (63%) and G‐CSF + PEF (53%) cohorts were approximately 20% lesser than reported literature [24, 26]. This could be because of longer lenalidomide exposure in these two cohorts (6 cycles). Similar to Japanese data [31], approximately 70% of our patients collected ≥ 5 million in Bort‐Cy‐G‐CSF in one apheresis. With respect to the Bort‐G‐CSF cohort, only 26% of our patients collected target cell dose ≥ 5 million, in contrast to that reported by Ghobadi et al. (≥ 6 million in 50%) [32]. However, 50% of their patients needed > 1 apheresis for achieving their target dose of 6 million and their pre‐mobilization regimens or lenalidomide exposure was not specified [32]. Amongst studies comparing G‐CSF‐PEF with Cy‐G‐CSF, considering a cut‐off of 4 million/kg, approximately 15% lesser patients achieve the target with the G‐CSF‐PEF regimen versus Cy‐G‐CSF [23, 25, 27], except in the Italian study by Lazlo et al. (90% in G‐CSF‐PEF arm) [27]. However, it should be borne in mind that none of the patients in the Italian study received lenalidomide prior to harvest [27].

Cyclophosphamide has been used at varying doses for PBSC mobilization—1.5‐2 g/m^2^ (low‐dose) [10, 25] or 3 g/m^2^ (intermediate dose) [24, 26] or 4 g/m^2^ (intermediate‐high dose) [23, 26, 27]. We have used Cy at a total dose of 2 g/m^2^ since 2007, as initial data showed similar efficacy and less toxicity with low‐dose Cy [33, 34]. The median CD34 cell dose of all harvests in our Cy‐G‐CSF (8.23 million/kg) and G‐CSF‐PEF (6.17 million/kg) group is comparable to western literature [10, 25, 27]. This is encouraging, as it suggests the efficacy of low‐dose Cy (2 g/m^2^) for PBSC mobilization in the Indian setting. The total CD34 cell dose in our Bort‐Cy‐G‐CSF group (9.2 million/kg) was much less than previously reported by Niesvizky et al. (23.2 million/kg) [21]. This could be because none of their patients were exposed to Lenalidomide [21], in contrast to 53% in our Bort‐Cy‐G‐CSF cohort. Moreover, all their patients underwent PBSC harvest within six cycles of treatment, in contrast to 27% in our Bort‐Cy‐G‐CSF cohort who had PBSC harvest at first relapse. Our median CD34 dose with Bort‐G‐CSF was similar to other small studies [30, 35]. Interestingly, similar to our findings, it appears from the literature that intravenous bortezomib [32] is better than subcutaneous for PBSC mobilization [30].

Our mobilization failure rates (cut‐off ≤ 2 million) in Cy‐G‐CSF (2%) and G‐CSF + PEF (3%) are comparable to literature [23, 25]. Notably, high failure rates of 18% with Cy‐G‐CSF in the study by Afifi et al. were probably because their cut‐off for defining “mobilization failure” was different (≤ 5 million) [24]. Our failure rates of 7% with Bort‐G‐CSF are unlike previous studies [30, 32, 35]. This discordance could be because 80% of patients in the Japanese study [30] received PEF, thereby undermining the effect of Bortezomib. In another study of Bort‐G‐CSF, 30% of patients needed 4 apheresis sessions to achieve the target CD34 of 6 million, while 20% required salvage apheresis with G‐CSF‐PEF in view of insufficient harvest [32]. Our Bort‐Cy‐G‐CSF arm did not have any mobilization failure, similar to literature [21, 31].

In patients who received VRd prior to apheresis (Table 5), we saw that the G‐CSF + PEF group is comparable to both chemo‐mobilization groups (Bort‐Cy‐GSCF and Cy‐GCSF) for the efficacy of stem‐cell harvest in the first apheresis. However, in Tables S2–, S5, it was evident that in patients who received prior RT to sites of hematopoiesis (vertebrae, pelvis), G‐CSF + PEF had an inferior yield (CD34 cell dose in first harvest—3 million/kg) and Bort‐Cy‐GCSF (CD34 cell dose in first harvest—5.32 million/kg) had the best yield in the first harvest. This knowledge is important as VRd is the most commonly used induction regimen in our setting. Hence, after this analysis, our institute policy has been to prefer the G‐CSF + PEF mobilization regimen, except for patients who received RT to sites of hematopoiesis (vertebrae, pelvis) wherein we prefer Bort‐Cy‐G‐CSF.

**TABLE 5:** Comparison of CD34 yield in all four groups in patients received VRd prior to harvest.

	Group 1—Bort‐GCSF (*n* = 43)	Group 2—GCSF‐plerixafor (*n* = 30)	Group 3—Bort‐Cy‐GCSF (*n* = 42)	Group 4—Cy‐GCSF (*n* = 90)	*p*
Patients who received VRd prior to harvest; *n* (%)	27 (63%)	18 (60%)	17 (40%)	16 (18%)	
Patients who collected ≥ 5 million in 1st harvest; *n* (%)	6 (23%)	10 (56%)	10 (59%)	13 (81%)	0.001 (Group 1 vs. 4)
CD34 cell dose in 1st harvest (×10^6^/kg); median (range)	3.62 (0.81–9.34)	5.76 (1.79–11.47)	5.61 (1.6–17.44)	5.72 (0.4–18.65)	0.04 (Group 1 vs. 2)
Total CD34 cell dose collected (×10^6^/kg); median (range)	6.05	6.7	6.8	8.8	0.03 (Group 1 vs. 4)
Median number of apheresis sessions; (range)	2 (1–2)	1 (1–2)	1 (1–2)	2 (1–4)	NS
Mobilization failure; *n* (%)	3 (11%)	1 (5%)	0 (0%)	1 (6%)	NS

Abbreviation: NS = not significant.

Our study has several limitations. This was a retrospective analysis over a 15‐year period with changes in the availability of induction regimen and choice of mobilization regimen, subject to the availability of generic medications like Lenalidomide and PEF. We have not addressed the adverse events and cost‐effectiveness among the four regimens. PEF subtraction analysis excluded patients who did not need PEF, thereby ruling out inherently good mobilizers. Importantly, our cohort's median age was 48 years, and less than 10% of patients were more than 60 years of age. Hence, the added value and comparison of chemo‐mobilization in elderly patients could not be ascertained. Our patients in different cohorts did not undergo apheresis at the same time point in induction. However, there was no difference in the median duration of lenalidomide exposure prior to harvest among the four groups. Newer drugs like Daratumumab and Motixafortide have been approved in myeloma as quadruplet therapy in newly diagnosed patients [6], and for stem‐cell mobilization [36], respectively. While Motixafortide is not available in India, Daratumumab is not feasible for the majority of the Indian population due to financial limitations. Our study primarily includes patients treated with bortezomib‐based triplet regimens. However, the strengths of our study include a large sample size, and no losses in follow‐up of the patients. This is the first study from India reporting various mobilization strategies for MM with a median follow‐up of more than 5 years. Moreover, our sample size for Bort‐G‐CSF (*n* = 43) and Bort‐Cy‐G‐CSF (*n* = 42) is larger than reported in pilot studies [21, 30, 31, 32, 35]. We have also compared various groups in patients treated with VRd induction, which is the current standard. Moreover, the effectiveness of different mobilization strategies has been studied in patients who have received prior RT and as per lenalidomide duration, which has given us new insights. Although not reported here, we recently published our group's incidence of second primary malignancies post‐ASCT in MM. Importantly, while our incidence of SPM was 3.3%, there was no difference in its incidence with respect to the mobilization regimen [37].

## 5 Conclusion

Cyclophosphamide‐based chemo‐mobilization regimens have the advantage of higher total stem‐cell yield, while they are equivalent to G‐CSF + Plerixafor for harvest in a single apheresis. There is no difference in engraftment rates and incidence of engraftment syndrome between chemo‐mobilization and other groups. The addition of Bortezomib to Cyclophosphamide may help to increase stem cell yield; however, bortezomib + G‐CSF is an inferior regimen.

## Author Contributions

**Sumeet Mirgh:** conceptualization, investigation, writing – original draft, methodology, validation, visualization, writing – review and editing, formal analysis, project administration, data curation, supervision, resources, software. **Bhausaheb Bagal:** conceptualization, writing – review and editing, data curation, supervision. **Sachin Punatar:** conceptualization, writing – review and editing, formal analysis, supervision. **Anant Gokarn:** writing – review and editing. **Nishant Jindal:** writing – review and editing, formal analysis. **Akanksha Chichra:** writing – review and editing. **Lingaraj Nayak:** writing – review and editing. **Sumathi Hiregoudar:** writing – review and editing. **Minal Poojary:** writing – review and editing. **Suryatapa Saha:** writing – review and editing. **Sarika Parab:** writing – review and editing. **Shashank Ojha:** writing – review and editing. **Prashant Tembhare:** writing – review and editing. **Nikhil Patkar:** writing – review and editing. **Sweta Rajpal:** writing – review and editing. **Gaurav Chatterjee:** writing – review and editing. **Libin Mathew:** project administration, data curation. **Papagudi Subramanian:** writing – review and editing. **Navin Khattry:** writing – review and editing, supervision, conceptualization, visualization, validation, project administration.

## Ethics Statement

This study (Project # 901095) was approved by the Institutional Ethics committee (Institutional Ethics Committee‐III ACTREC), with waiver of consent since this was a retrospective analysis without patient contact.

## Conflicts of Interest

The authors declare no conflicts of interest.

## Supporting information


**Data S1.** Per protocol analysis.

**Figure S1.** Survival curves as per Kaplan–Meier method. Upper left panel—PFS of entire cohort, upper right panel—OS of entire cohort, lower left panel—PFS of four groups, lower right panel—OS of four groups.

**Table S1.** Per‐protocol analysis of all four groups.

**Table S2.** Group 1 (Bort‐G‐CSF) performance with respect to prior radiotherapy (RT) and prior Lenalidomide (Len) exposure.

**Table S3.** Group 2 (G‐CSF‐Plerixafor group) performance with respect to prior radiotherapy (RT) and prior Lenalidomide (Len) exposure.

**Table S4.** Group 3 (Bort‐Cy‐G‐CSF) performance with respect to prior radiotherapy (RT) and prior Lenalidomide (Len) exposure.

**Table S5.** Group 4 (Cy‐G‐CSF) ‐ Performance with respect to prior radiotherapy (RT) and prior Lenalidomide (Len) exposure.

## Data Availability

For any further information, please send an e‐mail to the corresponding author.
