# Supplementary material for: Moving Beyond G‐CSF Mobilization—Learning From a 15‐Year Experience of Different Stem Cell Mobilization Regimens in Multiple Myeloma
Source: Cancer Med. 2025 Jul 16;14(14):e71068. doi: 10.1002/cam4.71068 (PMC12264575; doi:10.1002/cam4.71068)
Supplement: Supplementary file 1 — Data S1. Per protocol analysis. [file CAM4-14-e71068-s005.docx]

1. **Per-protocol analysis**

A separate per-protocol analysis was done (Supplemental Table 1), as eight patients in Bort-G-CSF group did not receive Bort on evening of day-4 as per clinician discretion. They received PEF, as they were deemed to be at high-risk of mobilization failure, as per CD34 counts (median CD34: 11.58/µL) on day-4 of G-CSF. Results were similar to ITT analysis.
