# Supplementary figures and images for: Moving Beyond G‐CSF Mobilization—Learning From a 15‐Year Experience of Different Stem Cell Mobilization Regimens in Multiple Myeloma
Source: Cancer Med. 2025 Jul 16;14(14):e71068. doi: 10.1002/cam4.71068 (PMC12264575; doi:10.1002/cam4.71068)

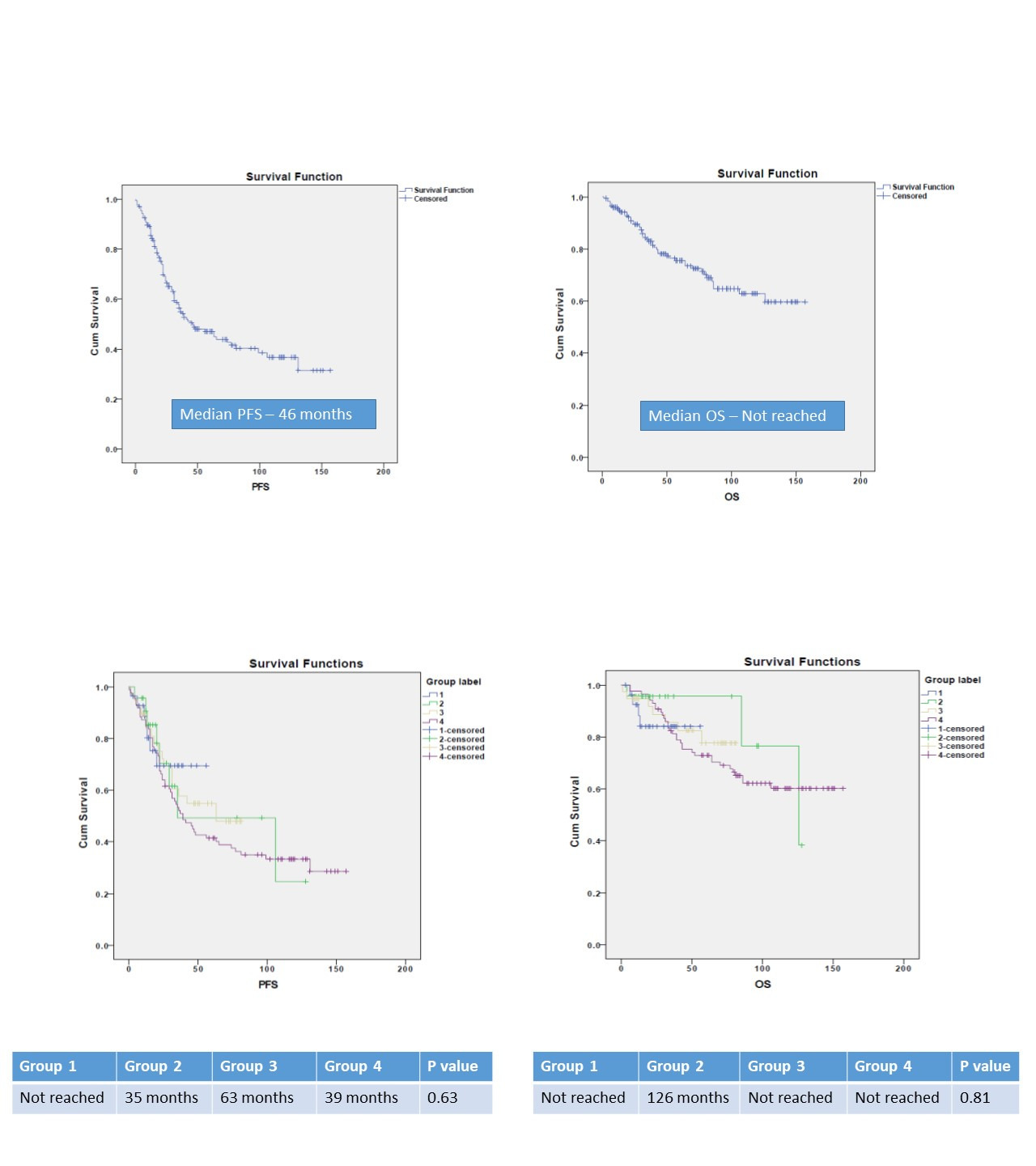

Supplement: Supplementary file 2 — Figure S1. Survival curves as per Kaplan–Meier method. Upper left panel—PFS of entire cohort, upper right panel—OS of entire cohort, lower left panel—PFS of four groups, lower right panel—OS of four groups. [file CAM4-14-e71068-s003.jpg]
