# Supplementary material for: Moving Beyond G‐CSF Mobilization—Learning From a 15‐Year Experience of Different Stem Cell Mobilization Regimens in Multiple Myeloma
Source: Cancer Med. 2025 Jul 16;14(14):e71068. doi: 10.1002/cam4.71068 (PMC12264575; doi:10.1002/cam4.71068)
Supplement: Supplementary file 3 — Table S1. Per‐protocol analysis of all four groups. [file CAM4-14-e71068-s004.docx]

**Supplemental Table 1 - Per-protocol analysis of all four groups**

| **Per-protocol analysis** | | | | | |
| --- | --- | --- | --- | --- | --- |
|  | Group 1 –  Bort-GCSF (n=35) | Group 2 –  GCSF-Plerixafor (n=38) | Group 3 –  Bort-Cy-GCSF (n=42) | Group 4 –  Cy-GCSF  (n=90) | P value |
| Patients who collected ≥5 million (1^st^ harvest); n(%) | 10 (28%) | 17 (45%) | 29 (69%) | 57 (63%) | 0.0001 (group 1 vs 3) |
| CD34 cell dose in 1^st^ harvest (x10^6^/kg); median (range) | 3.62 (0-11.61) | 4.43 (0-11.47) | 6.04 (1.6-17.44) | 6.05 (0.4-24.2) | 0.001 (group 1 vs 4) |
| Total CD34 cell dose collected (x10^6^/kg); median (range) | 5.73 (0-11.61) | 6.17 (0-15.9) | 9.143 (2.42-17.44) | 8.237 (0.4-24.2) | <0.00001 (group 1 vs 3) |
| Mobilization failure; n (%) | 2 (6%) | 2 (5%) | 0 | 2 (2%) | NS |
| **Plerixafor subtraction analysis – Analysis of patients who did not receive any plerixafor** | | | | | |
|  | Group 1 –  Bort-GCSF  (n=23) | Group 2 –  GCSF-Plerixafor | Group 3 –  Bort-Cy-GCSF (n=33) | Group 4 –  Cy-GCSF  (n=84) | P value |
| Patients who collected ≥5 million in 1^st^ harvest; n (%) | 9/23 (39%) | NA | 23/33 (70%) | 54/84 (64%) | 0.048 (group 1 vs 3) |
| CD34 cell dose in 1^st^ harvest (x10^6^/kg); median (range) | 4.77 | NA | 6.1 | 6.12 | 0.02 (group 1 vs 4) |
| Total CD34 cell dose collected (x10^6^/kg); median (range) | 6.03 | NA | 9.86 | 8.58 | 0.00007 (group 1 vs 3) |
| Mobilization failure; n(%) | 1 (4%) | NA | 0 | 1 (1.2%) | NS |

Abbreviations - Bort=Bortezomib, Cy=Cyclophosphamide, GCSF – Granulocyte colony stimulating factor, NA=Not applicable
