# Supplementary material for: Moving Beyond G‐CSF Mobilization—Learning From a 15‐Year Experience of Different Stem Cell Mobilization Regimens in Multiple Myeloma
Source: Cancer Med. 2025 Jul 16;14(14):e71068. doi: 10.1002/cam4.71068 (PMC12264575; doi:10.1002/cam4.71068)
Supplement: Supplementary file 4 — Table S2. Group 1 (Bort‐G‐CSF) performance with respect to prior radiotherapy (RT) and prior Lenalidomide (Len) exposure. [file CAM4-14-e71068-s001.docx]

**Supplemental Table 2 – Group 1 (Bort-G-CSF) performance with respect to prior radiotherapy (RT) and prior Lenalidomide (Len) exposure**

| **Groups – Exposure Yes vs No** | **Values** | **P value** |
| --- | --- | --- |
| **CD34 cell dose in 1^st^ harvest (in million/kg)** |  |  |
| RT – Yes (n=9) vs No (n=34) | 2.51 vs 3.67 | 0.06 |
| Len > 4 cycles – Yes (n=20) vs No (n=23) | 2.68 vs 3.91 | NS |
| **CD34 cell dose in all harvests (in million/kg)** |  |  |
| RT – Yes (n=9) vs No (n=34) | 4.75 vs 6.02 | 0.05 |
| Len > 4 cycles - Yes (n=20) vs No (n=23) | 5.57 vs 5.54 | NS |
| **≥5 million in 1^st^ harvest; %** |  |  |
| RT – Yes (n=1/9) vs No (n=10/34) | 11% vs 29% | NS |
| Len > 4 cycles – Yes (n=5/20) vs No (n=6/23) | 25% vs 26% | NS |

Abbreviations – Len=Lenalidomide, NS=Not significant, RT=Radiotherapy
