# Supplementary material for: Moving Beyond G‐CSF Mobilization—Learning From a 15‐Year Experience of Different Stem Cell Mobilization Regimens in Multiple Myeloma
Source: Cancer Med. 2025 Jul 16;14(14):e71068. doi: 10.1002/cam4.71068 (PMC12264575; doi:10.1002/cam4.71068)
Supplement: Supplementary file 5 — Table S3. Group 2 (G‐CSF‐Plerixafor group) performance with respect to prior radiotherapy (RT) and prior Lenalidomide (Len) exposure. [file CAM4-14-e71068-s006.docx]

**Supplemental Table 3 – Group 2 (G-CSF-Plerixafor group) performance with respect to prior radiotherapy (RT) and prior Lenalidomide (Len) exposure**

| **Groups – Exposure Yes vs No** | **Values** | **P value** |
| --- | --- | --- |
| **CD34 cell dose in 1^st^ harvest (in million/kg)** |  |  |
| RT – Yes (n=9) vs No (n=29) | 3 vs 5.13 | NS |
| Len > 4 cycles – Yes (n=18) vs No (n=20) | 4.16 vs 4.25 | NS |
| **CD34 cell dose in all harvests (in million/kg)** |  |  |
| RT – Yes (n=9) vs No (n=29) | 4.9 vs 6.23 | NS |
| Len > 4 cycles - Yes (n=18) vs No (n=20) | 6.11 vs 6.01 | NS |
| **≥5 million in 1^st^ harvest; %** |  |  |
| RT – Yes (n=2/9) vs No (n=15/29) | 22% vs 52% | NS |
| Len > 4 cycles – Yes (n=8/18) vs No (n=9/20) | 44% vs 45% | NS |

Abbreviations – Len=Lenalidomide, NS=Not significant, RT=Radiotherapy
