# Supplementary material for: Moving Beyond G‐CSF Mobilization—Learning From a 15‐Year Experience of Different Stem Cell Mobilization Regimens in Multiple Myeloma
Source: Cancer Med. 2025 Jul 16;14(14):e71068. doi: 10.1002/cam4.71068 (PMC12264575; doi:10.1002/cam4.71068)
Supplement: Supplementary file 6 — Table S4. Group 3 (Bort‐Cy‐G‐CSF) performance with respect to prior radiotherapy (RT) and prior Lenalidomide (Len) exposure. [file CAM4-14-e71068-s002.docx]

**Supplemental Table 4 – Group 3 (Bort-Cy-G-CSF) performance with respect to** **prior radiotherapy (RT) and prior Lenalidomide (Len) exposure**

| **Groups – Exposure Yes vs No** | **Values** | **P value** |
| --- | --- | --- |
| **CD34 cell dose in 1^st^ harvest (in million/kg)** |  |  |
| RT – Yes (n=16) vs No (n=26) | 5.32 vs 7.97 | 0.01 |
| Len >4 cycles – Yes (n=8) vs No (n=34) | 7.51 vs 5.81 | NS |
| **CD34 cell dose in all harvests (in million/kg)** |  |  |
| RT – Yes (n=16) vs No (n=26) | 8.89 vs 10.45 | NS |
| Len >4 cycles – Yes (n=8) vs No (n=34) | 7.99 vs 9.44 | NS |
| **≥5 million in 1^st^ harvest; %** |  |  |
| RT – Yes (n=9/16) vs No (n=20/26) | 56% vs 77% | NS |
| Len >4 cycles – Yes (n=5/8) vs No (n=24/34) | 63% vs 70.5% | NS |

Abbreviations – Len=Lenalidomide, NS=Not significant, RT=Radiotherapy
