# Supplementary material for: Moving Beyond G‐CSF Mobilization—Learning From a 15‐Year Experience of Different Stem Cell Mobilization Regimens in Multiple Myeloma
Source: Cancer Med. 2025 Jul 16;14(14):e71068. doi: 10.1002/cam4.71068 (PMC12264575; doi:10.1002/cam4.71068)
Supplement: Supplementary file 7 — Table S5. Group 4 (Cy‐G‐CSF) ‐ Performance with respect to prior radiotherapy (RT) and prior Lenalidomide (Len) exposure. [file CAM4-14-e71068-s007.docx]

**Supplemental Table 5 – Group 4 (Cy-G-CSF) -** **Performance with respect to** **prior radiotherapy (RT) and prior Lenalidomide (Len) exposure**

| **Groups – Exposure Yes vs No** | **Values** | **P value** |
| --- | --- | --- |
| **CD34 cell dose in 1^st^ harvest (in million/kg)** |  |  |
| RT – Yes (n=27) vs No (n=63) | 5.06 vs 6.72 | 0.05 |
| Len >4 cycles - Yes (n=18) vs No (n=72) | 4.34 vs 6.72 | 0.01 |
| **CD34 cell dose in all harvests (in million/kg)** |  |  |
| RT – Yes (n=27) vs No (n=63) | 7.02 vs 9.2 | 0.01 |
| Len >4 cycles - Yes (n=18) vs No (n=72) | 7.56 vs 9.0 | NS |
| **≥5 million in 1^st^ harvest; %** |  |  |
| RT – Yes (n=14/27) vs No (n=43/63) | 52% vs 68% | NS |
| Len >4 cycles - Yes (n=9/18) vs No (n=48/72) | 50% vs 66% | NS |

Abbreviations – Len=Lenalidomide, NS=Not significant, RT=Radiotherapy
